# Cultural adaptation of a children’s weight management programme: Child weigHt mANaGement for Ethnically diverse communities (CHANGE) study

**DOI:** 10.1186/s12889-019-7159-5

**Published:** 2019-06-28

**Authors:** Miranda Pallan, Tania Griffin, Kiya Hurley, Emma Lancashire, Jacqueline Blissett, Emma Frew, Paramjit Gill, Laura Griffith, Kate Jolly, Eleanor McGee, Jayne Parry, Janice L. Thompson, Peymane Adab

**Affiliations:** 1 Institute of Applied Health Research, University of Birmingham, Edgbaston, Birmingham, B15 2TT UK; 2 Department for Health, University of Bath, University of Bath, Bath, BA2 7AY UK; 3 School of Life and Health Sciences, Aston University, Aston Triangle, Birmingham, B4 7ET UK; 4 Warwick Medical School, University of Warwick, Coventry, CV4 7AL UK; 5 School of Social Policy, University of Birmingham, Edgbaston, Birmingham, B15 2TT UK; 6 Birmingham Community Healthcare NHS Trust, 1 Priestley Wharf, Holt Street, Birmingham, B7 4BN UK; 7 School of Sport, Exercise and Rehabilitation Sciences, University of Birmingham, Edgbaston, Birmingham, B15 2TT UK

**Keywords:** Childhood, Overweight, Obesity, Weight management, Ethnicity, UK

## Abstract

**Background:**

Childhood obesity prevalence continues to be at high levels in the United Kingdom (UK). South Asian children (mainly Pakistani and Bangladeshi origin) with excess adiposity are at particular risk from the cardiovascular consequences of obesity. Many community-based children’s weight management programmes have been delivered in the UK, but none have been adapted for diverse cultural communities. The aim of the Child weigHt mANaGement for Ethnically diverse communities (CHANGE) study, was to culturally adapt an existing children’s weight management programme for children aged 4–11 years so that the programme was more able to meet the needs of families from South Asian communities.

**Methods:**

The adaptation process was applied to First Steps, an evidence informed programme being delivered in Birmingham (a large, ethnically diverse city). A qualitative study was undertaken to obtain the views of South Asian parents of children with excess weight, who had fully or partially attended, or who had initially agreed but then declined to attend the First Steps programme. The resulting data were integrated with current research evidence and local programme information as part of a cultural adaptation process that was guided by two theoretical frameworks.

**Results:**

Interviews or focus groups with 31 parents in their preferred languages were undertaken. Themes arising from the data included the need for convenient timing of a programme in a close familiar location, support for those who do not speak English, the need to focus on health rather than weight, nutritional content that focuses on traditional and Western diets, more physical activity content, and support with parenting skills. The data were mapped to the Behaviour Change Wheel framework and Typology of Cultural Adaptation to develop an intervention programme outline. The research evidence and local programme information was then used in the detailed planning of the programme sessions.

**Conclusions:**

The process of cultural adaptation of an existing children’s weight management programme resulted in a theoretically underpinned programme that is culturally adapted at both the surface and deep structural levels.

**Trial registration:**

ISRCTN81798055, registered: 13/05/2014.

**Electronic supplementary material:**

The online version of this article (10.1186/s12889-019-7159-5) contains supplementary material, which is available to authorized users.

## Background

Childhood obesity is an ongoing public health problem in the United Kingdom (UK) with 20% of children aged 11 years experiencing obesity [1]. South Asian children in the UK experience even higher obesity levels (26 and 28% in 11 year old Pakistani and Bangladeshi children respectively [2]), and are more vulnerable to the cardiovascular consequences of adiposity both in childhood [3] and adulthood [4]. The last few decades have seen an exponential increase in childhood obesity, and alongside this, a number of behavioural programmes to assist children and families in managing their weight have been developed. More intensive, hospital clinic-based programmes have been offered for children with severe obesity, but in the UK, there has also been a focus on the development of community-based weight management programmes for children and their families, aimed at children with excess weight [5].

Systematic reviews and meta-analyses indicate that community-based weight management programmes for children result in a modest reduction in Body Mass Index (BMI) z-score (approximately 0.1 units 6 months post-intervention) [5, 6]. There is evidence that even very small reductions in BMI z-score can lead to lower cardiometabolic risk [7]. In the preadolescent age group, interventions that address both diet and physical activity, include behavioural elements, and involve parents have been shown to be the most promising [6, 8, 9].

Cultural adaptation is the process of developing interventions, based on pre-existing programmes and materials, that conform with the characteristics of the specified cultural communities [10]. There are few examples of cultural adaptation of children’s weight management programmes. Two USA-based Randomised Controlled Trials (RCT) which evaluated culturally adapted interventions, one targeting Chinese American children aged 8–10 years [11] and the other a mixed population of Hispanic, Black and White children aged 8–16 years [12], have reported small to moderate sustained reductions in BMI z-score in the intervention compared with the control groups. In the UK, one small RCT (*n* = 72) has been undertaken to evaluate the effectiveness of a family-based behavioural treatment programme, developed in the USA, targeting children with obesity in an ethnically and socioeconomically diverse community. The programme was not culturally adapted and did not have a significant effect on weight [13]. No culturally adapted interventions have previously been evaluated in the UK.

Theoretical approaches have been lacking concerning the process of cultural adaptation of both children’s weight management and health promotion programmes in general [14]. A theory-based approach to cultural adaptation of health promotion programmes is required, and the success of these adapted programmes needs to be evaluated by directly comparing adapted with standard programmes [15]. Retention of families in weight management programmes is important, as evidence suggests that better programme attendance leads to more weight loss [16]. Lower retention has been associated with certain programme characteristics (e.g. large group sizes [17]) but is also more common among children from certain minority ethnic families [18, 19], thus further highlighting the need for cultural adaptation of these programmes so that they better meet the needs of a wider range of families.

The aim of the first phase of the Child weigHt mANaGement for Ethnically diverse communities (CHANGE) study, was to culturally adapt a community weight management programme for primary school aged children. The programme selected to be adapted was a locally developed programme, incorporating elements of evidence-based child weight management programmes and taking into account the characteristics of the local population. Routine attendance data from this programme showed that it had poorer retention rates for children and families from Pakistani and Bangladeshi communities. Therefore, the purpose of the adaptation was to better meet the needs of families from Pakistani and Bangladeshi communities, thereby increasing their retention rates within the programme. This paper reports the process of cultural adaptation and the resulting adapted programme. Participant acceptability of and retention in the adapted programme has been evaluated in a subsequent feasibility study, which has been reported separately [20].

## Methods

### Setting

The study took place in Birmingham, the second largest UK city with a population of 1.1 million. Forty-two percent of all residents are from minority ethnic communities. Pakistani and Bangladeshi children comprise 26% of the Birmingham population aged 0–15 years [21]. At the time of the study a group-based child weight management programme, First Steps, was available across the city. The programme was delivered as weekly one-hour sessions over 5–7 weeks in community venues, covering nutrition education, physical activity promotion and the promotion of positive lifestyle behaviour changes. The programme was aimed at parents/carers; children attended only the first and last sessions to have their heights and weights measured. All families resident in Birmingham with a child aged 4–11 years with excess weight (BMI over the 91st centile of the UK 1990 growth reference charts [22]) and able to participate in a group setting were eligible to attend the programme. Children could be referred to the programme by a health professional, the child’s school, or families could self-refer. Children identified as having excess weight through the National Child Measurement Programme (a surveillance programme to provide data on weight indicators in primary school-aged children) were also referred to the programme.

### Programme selected for adaptation

First Steps was a children’s weight management programme developed by the service providers, based on their previous experience of delivering evidence-based programmes [23, 24], and tailored for the local population. Given Birmingham’s cultural diversity, there was a focus on parental engagement with access to interpreters, and programme material had a high pictorial content and referred to culturally appropriate foods. Despite this, Pakistani and Bangladeshi families who started the programme were less likely to complete it than families of other ethnicities (40% of Pakistani and Bangladeshi families completed it compared with 65% of families from other ethnic groups). Data collected routinely at the first and last sessions indicated that children achieved an average reduction in BMI z-score of 0.1 at programme end. This is in line with reported differences in BMI z-scores between intervention and control groups in randomised controlled trials of behavioural child weight management programmes [6]. Given the existing tailoring to the local population and evidence of effect on children’s weight, the programme provided a good foundation on which to develop a further culturally adapted programme, with the particular intention of increasing retention of families from Pakistani and Bangladeshi communities in the programme.

### Study design

The theoretical and modelling stages of the UK Medical Research Council (MRC) framework for the development and evaluation of complex health interventions [25, 26] guided the cultural adaptation process. The adaptation process was informed by three main information sources: 1) data from a qualitative study exploring the experiences and viewpoints of Pakistani and Bangladeshi families who had participated in or who had initially agreed but then declined to participate in the First Steps programme; 2) local information from the First Steps programme providers; and 3) existing children’s weight management literature. Two specific theoretical frameworks were used in parallel in the adaptation process: a framework for the development of behaviour change interventions, and a programme theory and adaptation typology to guide the adaptation of health promotion programmes for minority ethnic groups [15, 27]. An advisory panel comprising Pakistani and Bangladeshi parents of primary school-aged children also provided advice during the adaptation process. Ethical approval was received from the Edgbaston Local Research Ethics Committee in July 2014 (14/WM/1036).

### Qualitative study with Pakistani and Bangladeshi parents

Community Researchers from Pakistani and Bangladeshi communities in Birmingham with qualitative research experience (AA; female and of Pakistani heritage, and MB and SK; both female and of Bangladeshi heritage) were recruited to assist the core research team (TG (research fellow in Public Health with mixed methods research experience) and LG (lecturer in healthcare anthropology with extensive qualitative research experience); both female and of white British heritage) in undertaking this qualitative data collection. The Community Researchers did not have pre-existing relationships with participants prior to the study, but were able to communicate in Urdu, Bengali or Sylheti where necessary, and understand the cultural context of participating families.

The First Steps programme provider (Birmingham Community Healthcare NHS Trust) identified all Pakistani and Bangladeshi families who had been invited to take part in the programme from September 2013 to July 2014. The families were categorised into either: (i) attended 60% or more of the First Steps programme (‘completers’); (ii) started the First Steps programme but attended less than 60% (‘non-completers’); or (iii) did not attend the programme (‘non-attenders’). Parents from completing families were invited to participate in a focus group (FG) at a community venue. FGs were the preferred method of data collection as they explicitly use group interaction as a way of stimulating discussion [28]. However, we recognised that parents from non-attending and non-completing families may find it challenging to attend a FG, and so they were invited to participate in a one-to-one interview, which gave greater flexibility for them in terms of the timing and venue of the interview. Face to face interviews were preferred but telephone interviews were offered if this was not possible. We aimed to recruit 15 ‘non-completers’ and 15 ‘non-attenders’ to participate in interviews, and to hold 3–5 FGs with ‘completers’, with a contingency of recruiting more participants if data saturation was not felt to be achieved. All participants received a £10 shopping voucher following successful completion of the interview/FG.

Parents were initially contacted by telephone and a participant information pack was posted to those who expressed an interest in study participation. A further telephone call was made and if the parent agreed to participate, an interview or attendance at a FG was arranged. Parents who did not speak English were telephoned by a Community Researcher in their preferred language.

Interviews took place in the participant’s home and FGs at a convenient community location. Participants gave written informed consent and completed a short questionnaire before the interview or FG commenced. The interviews and FGs were conducted either by a core researcher or Community researcher in the participant’s preferred language. An additional researcher was present as an observer at the FGs. Semi-structured interview and focus group schedules, informed by literature and input from the study Parent Advisory Panel, were used to guide discussions. The research questions that were explored are shown in Table 1. Interviews and FGs were audio-recorded and transcribed. Community researchers translated and transcribed interviews and FGs that were not conducted in English. A sample of translated transcripts was checked using the audio-recording by an independent researcher with the relevant language skills.

**Table 1:** Research questions explored in phase 1 interviews and focus groups with Pakistani and Bangladeshi parents of overweight and obese children

Research question	
What are the participants’ experiences of the First Steps programme?	
What are the barriers and facilitators to participating in and completing the programme?	
Which aspects of the structure, content and delivery of the programme are perceived as problems?	
What aspects of the structure, content and delivery of the programme are valued?	
What information, content or format would increase the appeal of the programme?	
What might need to change about the current programme to ensure its cultural relevance?	

Data analysis was conducted using NVivo 10 (QSR International Pty Ltd. Version 10, 2012) and was guided by thematic analysis approaches [29]. Two researchers (TG and LG) reviewed 50% each of the transcripts independently and identified codes to apply to the data. The researchers discussed their coding and agreed on a final coding framework, which they then applied to all transcripts. Overarching themes were identified, which included commonalities and differences between the three participant groups.

### Information from the existing children’s weight management service

Direct observation of the First Steps children’s weight management programme was undertaken by a researcher (TG) to assess structure, content, delivery and participant response. In addition, a series of consultations were undertaken with the two service managers over a period of 3 months to enable an understanding of the existing infrastructure and processes. The managers were also asked to identify any issues with the existing programme from their perspective.

### Review of children’s weight management literature

A comprehensive guideline on managing overweight and obesity in children was published in 2013 by the UK National Institute for Health and Care Excellence (NICE) [5]. Two evidence reviews were undertaken to support development of this guideline, focusing on: 1) the effectiveness and cost-effectiveness of interventions to manage children’s weight [30]; and 2) the barriers and facilitators to implementing weight management programmes for children [31]. In addition a systematic review of behaviour change techniques that are effective in influencing obesity-related behaviours in children was published in 2013 [32]. These reviews, together with more recent evidence on effective children’s obesity interventions, informed the planning of the adapted programme to ensure that it was coherent with established evidence.

### Cultural adaptation process

The adaptation process was guided in parallel by two theoretical frameworks: The Behaviour Change Wheel (BCW) by Michie et al. [27, 33] and the Typology of Cultural Adaptation and Programme Theory of health promotion interventions by Liu et al [15]. The BCW has been developed from 19 behaviour change frameworks and was used to ensure the target behaviours, pathways to change, and adaptations made to address these were clearly articulated. Three target behaviours requiring change were identified; the first was programme attendance and the other two were behaviours that directly influence weight (dietary intake and physical activity). The capability, opportunity, motivation and behaviour (COM-B) model at the centre of the BCW enabled us to gain a theoretical understanding of the factors preventing Pakistani and Bangladeshi families from adopting the desired behaviours. This was achieved through the mapping of qualitative data from parents onto the different elements of the COM-B model (physical and psychological capability, physical and social opportunity, and reflective and automatic motivation). From this understanding of the factors influencing the target behaviours identified for change we were able to select the relevant intervention functions (categories of mechanisms by which interventions may have their effects) from the nine outlined in the BCW that correspond to elements of the COM-B model. This informed the detailed intervention planning.

The second framework, the Typology of Cultural Adaptation and Programme Theory proposed by Liu et al. [15], ensured that appropriate cultural adaptations were considered for inclusion in the adapted intervention across all aspects of the programme and at all stages of the programme cycle (i.e. conception/planning, promotion, recruitment, implementation, retention, evaluation, outcome, and dissemination). The 46-item typology has been constructed from a systematic review of health promotion programmes targeting smoking, diet and physical activity, which have been adapted for minority ethnic groups. The typology was used to identify the most appropriate type of cultural adaptations to address the themes identified in the qualitative data obtained from Pakistani and Bangladeshi parents.

### Detailed intervention planning

**Fig. 1:**
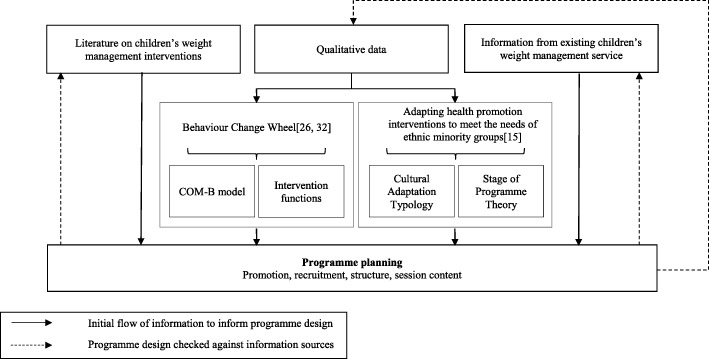
Process of cultural adaptation of a child weight management programme

The identified BCW intervention functions and the types of cultural adaptation provided the outline for the detailed planning of the adapted programme. The local information from direct observation and the service providers, and the relevant literature were used to further inform the process. Consideration was also given to the flexibility of programme delivery to ensure suitability for children of different ages. The planning process was iterative to ensure that the final programme design was coherent with: a) the identified intervention functions and adaptation types; b) the qualitative data; c) local service information; and d) the children’s weight management literature. Figure 1 summarises the intervention adaptation methodology.

## Results

### Findings from qualitative study with Pakistani and Bangladeshi parents

In total, 31 parents/carers participated in interviews and 12 participated in FGs. All participants were Muslim, 36 (84%) were Pakistani and 37 (86%) were female. Twenty-one participants were ‘non-attenders’, 9 were ‘non-completers’ and 13 were ‘completers’. Participant characteristics are shown in Table 2.

**Table 2:** Demographic characteristics of the 43 parents participating in the study

	Completers	Non-completers	Non-attenders	All participants
	(*n* = 13)	(*n* = 9)	(*n* = 21)	(*n* = 43)
Sex, n (%)				
Male	3 (23.1)	2 (22.2)	1 (4.8)	6 (14.0)
Female	10 (76.9)	7 (77.8)	20 (95.2)	37 (86.0)
Age of child ^a^, median (IQR)	11.0 (2.0)	11.5 (3.0)	11.0 (6.0)	11.0 (3.0)
Sex of child referred to the programme (*n*)^a^				
Male	7	5	8	20
Female	7	5	14	26
Relationship to the child, *n* (%)				
Mother	10 (76.9)	7 (77.8)	20 (95.2)	37 (86.0)
Father	3 (23.1)	2 (22.2)	1 (4.8)	6 (14.0)
Ethnicity, *n* (%)				
Pakistani	12 (92.3)	8 (88.9)	16 (76.2)	36 (83.7)
Bangladeshi	1 (7.7)	1 (11.1)	5 (23.8)	7 (16.3)
Referral method, *n* (%^b^)				
Doctor	0 (0.0)	2 (22.2)	1 (4.8)	3 (7.0)
School Nurse	2 (15.4)	0 (0.0)	3 (14.3)	5 (11.6)
NCMP	9 (69.2)	4 (44.4)	12 (57.1)	25 (58.1)
Hospital/dietician referral	1 (7.7)	1 (11.1)	2 (9.5)	4 (9.3)
Leaflet/self-referral	1 (7.7)	2 (22.2)	3 (14.3)	6 (14.0)
Method of discussion, *n* (%)				
Interview	1 (7.7)	9 (100.0)	21 (100.0)	31 (72.1)
Focus group	12 (92.3)	0 (0.0)	0 (0.0)	12 (27.9)

^a^1 completer, 1 non-completer, and 1 non-attender had two children who attended or were referred to the programme

^b^Percentages may not sum to 100 due to rounding

Of the 31 interviews, 27 were conducted face to face and 4 by telephone. Six interviews were in Urdu and 3 in Bengali. Length of interviews ranged from 15 to 47 min (average: 28 min). Once interviewees seemed to have no further comments, interviews were drawn to a close. Four FGs were completed. A further 3 were arranged but no participants attended. Two of the FGs were attended by 4 participants with the remainder having 2 participants. Two FGs were conducted in Urdu. The FG length ranged from 35 to 50 min.

Several themes emerged from the data. There was coherence across the three groups on several themes, but some themes were more prominent in some groups than others. Important logistical barriers to attending a family community weight management programme were raised by all participants. The majority of families reported that to attend the programme it would need to be in a close, familiar location at a convenient time. Some parents were concerned about children missing school and identified weekends as the most convenient time to attend, whilst others felt that children could take time out of school to attend. In contrast, after school sessions were commonly considered to be impractical due to many of the children attending religious classes at their local mosque at this time. This practice was also raised as a barrier to finding time for being physically active. Caring for younger siblings was cited as a barrier to attending by some parents, although it was also observed that younger siblings were often brought along to the sessions. Language barriers to participation existed for some parents from Pakistani and Bangladeshi communities who did not speak English. These were highlighted as a problem by some non-attenders at the initial recruitment stage. Once participants attended the programme, language barriers were less of an issue, particularly if interpreters were present (all participants were asked whether they required an interpreter prior to commencing the programme). Several English speaking participants discussed supporting other parents in the group who were struggling to understand.

The focus of the programme being on weight and obesity, rather than a positive focus on health was also a barrier. Some parents, particularly those who had not attended or completed the programme, considered that their child did not have a weight problem, or felt that they could not do anything to address their child’s weight. These families engaged less with the programme as its focus was on weight loss. However, data from these parents indicated that they recognised the value of healthy lifestyles and wanted to encourage their children to adopt healthy behaviours. Some parents who did not attend or complete the programme also highlighted that children were sensitive about attending for “weigh ins”.

Another group of important themes related to the target audience, content and delivery of the programme. Most parents felt that a programme involving children in all the sessions would be of more value, as they felt that children need to learn how to change their behaviour first hand, and would respond more positively to messages relating to behaviour change if they were given by someone other than their parents. Interactivity within the programme was highlighted as important. Completing participants spoke of the value of the interactive elements of the programme. However, non-completing participants perceived that there was little interactive content and disliked the ‘classroom’ format of sessions. They also reported that they disliked receiving a high volume of written information. Many of the participants expressed that there needed to be much more physical activity content in the programme, particularly getting the children to participate in physical activities during the sessions. They identified a range of barriers to physical activities in their daily lives which they thought should be addressed through the sessions. The group setting and the ability to share ideas and experiences amongst attending families was highly valued by many participants who had attended the programme. Some participants who had not attended or completed the programme felt that they were not going to gain anything new from it, and that they already had a good idea what was ‘good’ and ‘bad’ for their children, particularly in terms of their diet. This viewpoint differed in several of the completing participants, who felt they had gained new nutritional knowledge, and also advice on how to apply this in their daily lives. Although the First Steps programme included references to South Asian foods, some parents felt that the nutritional content could be made more relevant to their traditional diets, whilst other participants acknowledged the importance of also talking about Western foods, as their children’s diets encompassed both traditional and Western foods. There were mixed views regarding the cooking methods of traditional foods; some participants felt there was opportunity to learn about healthier cooking methods (e.g. using less oil), but others felt that they would not change their cooking methods. There was also concern about children’s intake of ‘junk’ foods, which they felt needed to be addressed. Finally, several parents who had attended part or all of the programme expressed difficulty in ensuring their children adhered to the changes that they instigated at home, especially in relation to food, and therefore, they felt they needed help with overcoming this issue.

Apart from dietary and language factors and the time spent attending religious classes, no other emerging themes related explicitly to Pakistani and Bangladeshi culture. The more prominent issues identified in the data were the difficulties and competing priorities which families had to face in their daily lives (e.g. juggling demands of siblings, busy family lives, and perceived safety issues in local communities), and the impact of these on the ability to undertake healthy behaviours. The emergent themes and examples of data to illustrate these themes are shown in Table 3.

**Table 3:** Themes emerging from the interviews and focus groups with Pakistani and Bangladeshi parents, and quotes to illustrate the themes

Themes	Completers	Non-completers	Non-attenders
Logistical issues with programme attendance			
Close location	*If it’s closer, then it’s better because it saves time; because sometimes we have to collect the children, and both mother and father needed to attend, so we both went.* (FG3 (conducted in Urdu), P2, male, Pakistani)		*Well, it shouldn’t be too far away, it’s better if it’s closer because sometimes the car isn’t available and then I could walk too.* (154, female, Pakistani (interview conducted in Urdu)) *The reason why I couldn’t make it is because I’m not driving, so having to travel to the place and then coming back with another small child, at the time I think she was a baby, was really difficult for me… It was just that really, I really want to go as well* (144, female, Pakistani)
Familiar venue	*I think if you go through the school it’s better. Everybody has to take their children to school. So if in the morning when they’ve gone to school to drop their child off or in the afternoon if the teachers come forward and talk to the parents then like ‘this is what’s happening and if you would like to attend’ maybe they would be, because everybody takes their kids to school and that would be a good way of catching them.* (FG1, P3, female, Bangladeshi)		
Programme timing	*I think that it’s about timing because some people have young children and others are older so they need to pick them up from school, others are in college so they need to collect them, so I think it’s about timing.* (FG3 (conducted in Urdu), P1, female, Pakistani)		*…the timing and, you know, it’s not – and town is like, you know, busy and…so…especially after 4. It’s really hard. They have, like, their own activity. Mosque and everything. Tuition. This and that. So that’s why I couldn’t* (133, female, Pakistani) *Weekends, because after school they go to school and Mosque, all Muslims, even Indian or Bengali or Pakistani, every Asian, children attends Mosque after school* (139, female, Pakistani)
Programme in school time	*Well, you know this weekend, it would be better, the children would be home and you could take them instead of missing them and they’re taking time off from school.* (FG1, P1, female, Pakistani)	*I’m sure if it’s a school day, the school would give him an hour or so just to go into, it’s regarding health isn’t it, so I’m sure school would allow him to go for an hour or do the programme in the weekend like Saturday/Sunday.* (129, male, Bangladeshi)	*I was upset because I couldn’t go. I couldn’t have the time, I couldn’t take my – especially with schools now where they’re strict on the children, you know, attending school and not missing days. So it was hard for me.* (104, female, Pakistani)
Siblings	*I didn’t have younger children but other families had young children with them. And they sat too, it wasn’t that the younger ones couldn’t sit and listen too*. (FG2, P4, female, Pakistani)		*When I started receiving letters and phone calls from yourselves then I realised that there might be support. My daughter says to me that ‘mamma I want to go for exercise’... I told her that I couldn’t go with her because I have other children. I have small children, my youngest is 2 years old.* (109, female, Pakistani (interview conducted in Urdu))
Language barriers			
Initial contact			*Someone rang on my home phone speaking English & inviting me to attend the programme but I was asking her if I needed to take my daughter with me, because my English is not very good; but she could not understand what I was trying to ask her. I was asking if I needed to take my daughter with me. She couldn’t understand me so she said she will call me back but we never heard from her again.* (150, female, Bangladeshi (interview conducted in Bengali))
Programme sessions	*I don’t know English, they were English, but I understood everything because of the way they explained it, with gestures and all the information so that we could understand.*(FG3 (conducted in Urdu), P2, male, Pakistani) From FG3 (conducted in Urdu): Facilitator: *So was there a translator there?* Participant 1: *No. Because at first I didn’t really mention it because my daughter was with me and so I didn’t have any problems because my daughter would speak for me and she’d translate what I was saying back to them about what to do* etc. (female, Pakistani)	*Yes because I’ve seen some parents there that are like it was hard for them to understand and I was doing a lot of explaining to them as well* (123, female, Pakistani)	*My niece had taken her son to First Steps programme, but she herself didn’t understand English, right? … She told me what was there, but she felt left out, as a parent—saying that, you know, ‘If there’s enough information for me, because I can’t read,’ she said, ‘and I can’t understand, then it would have been easier if there was somebody to explain to me* (113, female, Pakistani)
Programme structure and delivery			
Programme and session duration			*It’s not reasonable for me going and going back and coming back, so that is an issue, as well. So if the hours were extended, like an hour and a half or two hours, that would be reasonable.* (113, female, Pakistani) *I think 7 weeks is OK, to be honest, yeah. That’s not a problem. I think that’s just about right to be honest, yeah. Because if you make it too long, probably get a bit boring wouldn’t it.* (143, female, Pakistani)
Children attending	*Because sometimes children don’t listen to their mum or dad but they listen to the teacher or outsider* (FG4 (conducted in Urdu), P1, male, Pakistani) *So if like you know if like if these sessions are done but then it’s explained to the kids a little bit more about ‘this is what you need to do because it’s your life, you’re going to be affected in the future’ and stuff like that then it might help them*. (FG1, P3, female, Bangladeshi)	*It would have been a bit more ideal if the kids were more involved. That’s what I would -, because then yes we need to have that understanding, but I believe the kids need to understand what they should have and the intake and how it’s with their body.* (107, female, Pakistani) *I think children should go every session because then, you know, well how I look at is if the children don’t go and then we’re telling ‘oh you’ve got to do this, this’, they probably think we just sometimes, most kids, they will think oh just my parents being horrible to me, my parents, but when they go into classes and they see these other people they don’t know who are actually telling them, then they will listen more because they will think: hang on if I don’t know the chap there was telling me, so I think my dad is right, so yeah OK I’ll try that.* (129, male, Bangladeshi)	*...although my daughter does listen to me. I think getting the information first hand would make a big difference. So it’s important for both mother & child to attend.* (150, female, Bangladeshi (interview conducted in Bengali))
Programme interactivity	*The visual, it was the visual things really that she all brought the visual things and that really like makes it more better understanding then like you know.* (FG1, P1, female, Pakistani) [Participant talking about a related workshop that was not delivered as part of the main programme] *It wasn’t really cooking it was just readymade wraps, and you would just put salad in it, and we needed to cut it and put it in and whatever you need to put in there like butter they had brought along with them. So we cut it up, and the children cut it up and made them and then you have a look. In this way I think the children enjoy it too, so they understand that this is happening for them, so it sinks into their minds that if they do this then it will be of benefit to them.* (FG3 (conducted in Urdu), P1, female, Pakistani)	*I thought it was going to be like kind of activities where they actually show you what kind of activities you can do with your children, what kind of sports and obviously get them interested in them kind of activities. But obviously it was like just basically information just sit there and obviously giving us information about what kind of nutrition and diet and exercise and everything but I thought it was going to be more physical than obviously classroom based* (142, female, Pakistani) *I think there was a bit too much paperwork and what it is, she was giving out the information, yes she was trying her best, but I think the way she was delivering it everyone was like going half asleep... because some parents don’t take it in as that, and it’s like they need to get up and do* (107, female, Pakistani)	
Group sessions and shared experiences	*There was different community families, and friendly. Indian, Bengali, English, Sikh, and children’s mix up, and share their experiences.* (FG2, P4, female, Pakistani) *Because it was the same lady* [facilitator] *for all five sections, and she nicely laughs and you know and mostly my son was happy you know and when different communities people sit and talk and like it was like a challenge between everyone and she used to push them to compete.* (FG2, P4, female, Pakistani)		*I think this is a really good idea like when you go to a talk then you get to hear the views of others and that has an effect on you* (109, female, Pakistani (interview conducted in Urdu))
Programme content			
Focus on weight status		*I know it was weigh in and there was less time but with the kids I think if they approach them a bit differently because nowadays kids are very, very sensitive and every sort of thing just sticks in their head and I think, you know, ‘oh God mum’ and then in school they’ll have that -, because they had to come out of school and then it’s them like ‘oh we’re going for the weigh in’ and she was embarrassed to even tell her brother and sisters what she was going for* (107, female, Pakistani)	*I don’t see it as overweight, ‘cause I know what they eat. I know they’re not eating the wrong food. Yes, they’re less active, but what do you do?* (108, female, Pakistani) *My daughter, she’s not really overweight, it’s just that her weight has gone a bit over the mark* (104, female, Pakistani) *I mean, if you look at my son, he’s not overweight, I mean, he’s quite, for his age, he looks bigger than his age, I mean, he doesn’t look like really big or anything but he is quite heavy* (144, female, Pakistani)
Nutritional knowledge and skills	*But the way they explained everything it was very interesting. I didn’t know just a bottle of water with lemon juice had like so many rounds of sugar in there and all that stuff and like they said biscuits you think that’s the healthy option, actually it isn’t. You know like so it was quite an eye opener.* (FG1, P3, female, Bangladeshi) *Because they brought a lot of material about foods with them, like sweet packets, crisps, sugar* etc.*, all these things were there and how much sugar was in them. How much salt is in things and how to swap these things and it will be effective. And I did this 100% and it took effect.* (FG3 (conducted in Urdu), P1, female, Pakistani)	*We went on the first session. The minute that plate came up and those sugary – you know, those little packets and everything, we thought, ‘Oh, we’ve been there, done that. Forget this’* (121, female, Pakistani) *When you buy the shopping, more labelling, more information, because I understand what they say sometimes there’s energy and then the parents, some get confused because obviously and some English is not even there, so if they can like give a bit more which is more better and which is more healthy, like* [drink brand]*, because I didn’t pick it up from there,* [drink brand] *does have a lot more sugar than we thought* (107, female, Pakistani)	*I thought it would be just like talking through healthy and unhealthy but myself, I always look on the internet for healthy options, healthy meals and you know what’s good for me, what’s not good for me. So I’m constantly on the internet, right? So I thought I probably know it anyway’* (104, female, Pakistani)
South Asian and Western foods	From FG1: Interviewer: *And what sort of foods would you like to learn about in cooking, westernised or traditional or a bit of both?* Participant 4: *A bit of both, yeah.* Participant 1: *A bit of – the children do have both.* (female, Pakistani) Participant 4: *They get to have, they get bored with this type of food all the time, they want to try something different. So that would be like a mixture really*. (female, Pakistani)		*I think they should talk about both* [Asian and Western food]. *We do eat Asian food a lot but my children like both so it would be beneficial to get advice on both.* (150, female, Bangladesh (interview conducted in Bengali)) *We do eat fish and we do eat baked beans and stuff, but we do eat our own food, as well, so we need education on our own food* (113, female, Pakistani) *We eat a range of foods and my daughter likes eating food like this. They eat Pakistani food too but also English foods that are vegetarian.* (109, female, Pakistani (interview conducted in Urdu))
Cooking of traditional foods	*Yeah because if I change using less oil, I can’t taste my curry without oil, since I was 3 and have grown up, I can’t change that but I can swap other things, fat milk with semi skimmed and white with wholemeal breads but I can’t change my curries.* (FG2, P1, female, Pakistani)		*I want to know, if I’m making a chapatti, how many calories are in there? You know. If I’m making a curry – it’s really hard to – how many calories – you know, hand-size or, you know, it’s hard – in reality, it’s really, really hard. Maybe do a cooking session; say, ‘This is a portion.’ You know. ‘It’s right.’ Maybe do it that way…or even, like, give recipes on maybe even healthier Asian food, rather than – fair enough, do the English food, as well. OK, we have it once a week or whatever. And that’s ovenly – oven-made or it’s grilled. But help us with the type of food that we’re eating. Where are we going wrong?* (108, female, Pakistani)
‘Junk’ foods and takeaways			*She eats a lot of chocolates, sweets and crisps, she eats a lot of takeaways, like burgers, drinks a lot of fizzy drinks, she eats a lot of this stuff. Stuff like chapatti and curry, she eats less of.* (154, female, Pakistani (interview conducted in Urdu)) *But, the temptation in this area is that we have cheap takeaways, and they are very tempting. You know, you think, ‘Why cook?’ And, you know, we’re tempted to, you know, just, ‘Oh, it’s an easier option. We’ll get chicken and chips. It’s only £1.50.’ So, you know, that’s why the weight is creeping up with children* (113, female, Pakistani)
Physical activity content	*If they could like have a meeting for half an hour and then integrate like another half an hour to do the sports, I think that would be good as well.* (FG1, P3, female, Bangladeshi) *I think that if you are doing this programme then you need to put some exercise sessions in it too, whatever is best for children... if you have the space then you should have exercise programmes in it too* (FG3 (conducted in Urdu), P1, female, Pakistani)	*They should do more activities like, you know, physical activities to help them and not just concentrate on the food side* (123, female, Pakistani*)*	
Barriers and facilitators to physical activity	*And you can do something at home as well, children sitting down, it’s better to tell them to walk like ten times on the stairs, up and down. That’s a good exercise for them.* (FG2, P1, female, Pakistani)	*There’s just nowhere for us to send them where they can get exercise. Whether they can play football or cricket or anything, they should do something. And I would enrol them there.* (155, female, Pakistani) *My sister gets into the car and drops them off to the secondary school, you see. But they need that exercise. They need to learn how to walk, as well. You know, the car is very convenient, but it’s really bad for the kids* (121, female, Pakistani)	*We rarely get to go to the park unless it’s a hot summer’s day. It’s just busy.* (108, female, Pakistani) *I want to ride a bike ... and my husband goes ‘can you see how dangerous it is, the cars out there* (112, female, Pakistani) *And you can’t let them go to the parks alone. And it’s just round the corner but you just can’t... You just can’t let them out, ‘cause a lot has been, you know, happening around here.* (108, female, Pakistani)
Parental behaviours and influence over child	*I’ve tried to cut down. You know they showed us a certain plate of vegetables, that’s how much and all that stuff and I’ve tried doing that, I’ve really tried getting into it but I find that he sneaks behind me, he goes in the kitchen and helps himself.* (FG1, P3, female, Bangladeshi)	*When he goes to my mum’s house, he helps himself a lot and then when we go to family, like, he doesn’t listen, he helps himself a lot* (103, female, Pakistani) *But the drink wise, he does drink sometimes fizzy drink and I’m going to deny that I do bring sometimes, I feel bad, they like it, right, so just drink a bottle and give it to them, I say ‘look hide it* (129, male, Bangladeshi)	

### Findings from review of children’s weight management evidence

The UK NICE guideline on managing overweight in children and young people (PH47) [5], published in 2013, presented several evidence-based recommendations regarding children’s weight management service provision. The recommendations were taken into consideration during the detailed planning phase of programme adaptation to ensure that the finalised programme was consistent with the guideline (see Table 4). The guideline, along with other relevant literature [5, 34], emphasised the importance of parental involvement in child weight management programmes, and the need for elements that address both diet and physical activity [35, 36]. Therefore, these important aspects were included in the adapted programme. The behaviour change techniques that have been identified as effective in obesity interventions for children [32] (provision of information on the consequences of behaviour to the individual; environmental restructuring; prompting practice; prompting identification of role models or advocates; stress management/emotional control training; and general communication skills training) were also considered for inclusion in the adapted programme.

**Table 4:** Mapping of qualitative themes to COM-B components and cultural adaptation types, identification of intervention functions, planned intervention design and corresponding NICE recommendation

Factors to address identified from qualitative data	Behaviour Change Wheel		Cultural adaptation		NICE guidelines^b^	Intervention adaptation
	COM-B element	Intervention function	Typology of adaptation^a^	Programme theory stage		
Behaviour target 1: Improve session attendance and completion of the programme						
Convenient programme location Ease of travel and parking Convenient timing of programme	Physical opportunity	Environmental restructuring	25. Consider target populations employment/home situations 29. Utilise appropriate incentives and timing of programme 33. Located in ethnically/culturally appropriate/familiar location	Conception/ planning Promotion Recruitment Retention	Programmes should be provided at flexible times to meet the needs of the community	Increase opportunity for Saturday sessions Identify convenient programme locations (e.g. schools, good transport links)
Parental responsibility for other siblings	Physical opportunity Psychological capability	Environmental structuring Enablement	24. Intervention delivered in a culturally appropriate or preferred format 39. Address structural barriers to participation	Promotion Recruitment Retention		Allow siblings to attend Ensure siblings are made welcome and included in sessions
Facilitate children attending in school hours	Psychological capability	Enablement	38. Address emotional barriers and stressors	Promotion Recruitment Retention	Programmes should provide a tailored plan to meet the needs of the child and family (such as child age, family social and economic circumstances, ethnicity, and cultural background)	Improve knowledge of authorisation for children to have time out of school
Language barriers at initial recruitment Language requirements in programme sessions	Psychological capability Social opportunity Reflective motivation Automatic motivation	Enablement	14. Reflect target population’s language	Recruitment Implementation Retention Evaluation		Provide high quality language support at recruitment stage and within programme
Increase duration of programme sessions	Physical opportunity	Environmental restructuring	24. Intervention delivered in a culturally appropriate or preferred format	Conception/ planning Implementation	–	Increase session length from 60 to 90 mins
Weight not perceived as a) a problem or b) something that can be changed by some parents	Reflective motivation Automatic motivation Psychological capability	Education Persuasion Enablement	22. Intervention content targets population’s social and cultural values 23. Intervention goals and outcomes are culturally appropriate	Conception/ planning Promotion Recruitment Implementation Outcome	Programmes should be multicomponent and focus on diet, healthy eating habits, physical activity, reducing time spent sedentary and strategies for changing behaviour of the child and their family	Focus on the benefits of healthy behaviours for good health outcomes at recruitment and throughout the programme (vs. focus on weight) Inclusion of effective behaviour change techniques
Sensitivity of children to being weighed	Automatic motivation	Enablement	38. Address emotional barriers and stressors	Conception/ planning Recruitment Implementation Outcome		Focus on healthy behaviours to influence health outcomes, rather than weight
Interactive format better received than didactic format	Social opportunity Automatic motivation	Enablement	16. Reflect target population’s preferred method of communication 24. Intervention delivered in a culturally appropriate or preferred format	Conception/ planning Implementation Retention	Programmes should include behaviour change techniques parent skills training, incorporate learning of practical skills and introduce simple physical activity opportunities within the programme	Inclusion of more interactive activities More opportunities to socialise and share experiences to encourage peer support
Visual materials are important to communicate messages	Psychological capability Automatic motivation	Education Persuasion		Conception/planning Implementation Retention		Inclusion of visual materials with clear educational messages
Parents prefer less ‘paperwork’ (handouts)	Psychological capability Automatic motivation	Education Environmental restructuring	15. Match reading level and literacy 16. Reflect target population’s preferred method of communication	Implementation Retention	Programmes should provide a tailored plan to meet the needs of the child and family (such as child age, family social and economic circumstances, ethnicity, and cultural background)	Reduce volume of handouts; make them attractive and visual, with less written information
Children should attend all sessions to interact directly with programme facilitators	Physical opportunity Social opportunity	Environmental restructuring	24. Intervention delivered in a culturally appropriate or preferred format	Conception/planning Promotion Recruitment Retention		Children attend all sessions with parents Session content appropriate for children aged 4–11 years
Encourage social interaction and peer support	Social opportunity Automatic motivation	Enablement	41. Encourage/ involve social support	Conception/ planning Implementation Retention		Inclusion of more interactive activities More opportunities to socialise and share experiences to encourage peer support
Perceived value of the programme; parents feel they have enough knowledge about healthy lifestyles	Reflective motivation	Education Persuasion Incentivisation	19. Material/guidance based on preferences of target population 23. Intervention goals and outcomes are culturally appropriate	Conception/ planning Recruitment Implementation Retention Outcomes	Include parent skills training, behaviour change techniques and learning of practical skills	Increased focus on how to change dietary and physical activity behaviours Inclusion of effective behaviour change techniques Attractive recruitment materials, emphasising relevance of programme to families
Behaviour target 2: Improve physical activity behaviours						
Physical activities should be included in the sessions	Physical opportunity	Training Enablement	19. Material/ guidance based on preferences of target population 36. Provide ethnically/culturally appropriate food/activities	Conception/ planning Recruitment Implementation Retention Outcome	Programmes should introduce simple physical activity opportunities within the programme	Incorporate fun physical activities into all programme sessions
Lack of local physical activity opportunities, lack of time for physical activity and reliance on sedentary transport	Physical opportunity Psychological capability	Education Training	22. Intervention content targets population’s social and cultural values 24. Intervention delivered in a culturally appropriate or preferred format 25. Consider target populations employment/home situations 36. Provide ethnically/culturally appropriate food/activities 39. Address structural barriers to participation	Conception/ planning Implementation Retention Outcome	Programmes should provide a tailored plan to meet the needs of the child and family (such as child age, family social and economic circumstances, ethnicity, and cultural background)	Include a range of physical activities throughout, led by the facilitator, encouraging simple movement patterns and aerobic exercise opportunities that can be performed in the home and require little time Address cultural norms resulting in perceived limitations to physical activity Discuss active transport and other walking opportunities
Perceived dangers of undertaking physical activity	Psychological capability Automatic motivation	Training Modelling	38. Address emotional barriers and stressors 39. Address structural barriers to participation	Implementation Retention	Programmes should introduce simple physical activity opportunities within the programme	Undertake fun and safe physical activities that can be done at home
Parents’ perceived ability to effectively influence their child’s physical activity behaviours	Psychological capability	Enablement Training	23. Intervention goals and outcomes are culturally appropriate 26. Intervention addresses health behaviour patterns found in target populations 38. Address emotional barriers and stressors 41. Encourage/involve social support	Conception/ planning Implementation Retention Outcome	Programmes should include behaviour change techniques to increase confidence and motivation in ability to make changes and also include parent skills training	Improved social support to encourage self-belief Encourage parental physical activity Incorporate parenting skills training Set achievable targets and rewards
Behaviour target 3: Improve dietary habits						
A need to address both Asian and Western foods in sessions focusing on diet	Reflective motivation Social opportunity	Education Enablement	19. Material/ guidance based on preferences of target population 27. Dietary issues unique to their context 36. Provide ethnically/culturally appropriate food/activities 43. Maintaining cultural significance of food	Conception/ planning Implementation Retention	Programmes should provide a tailored plan to meet the needs of the child and family (such as child age, family social and economic circumstances, ethnicity, and cultural background)	Nutrition education content to include traditional and Western food examples Sensitivity to the social importance of food in different cultures Encourage sharing of skills and experiences through social interactivity and support
A need to know how to prepare healthier food	Physical capability	Training	24. Intervention delivered in a culturally appropriate or preferred format 36. Provide ethnically/culturally appropriate food/activities 43. Maintaining cultural significance of food	Conception/ planning Implementation Outcome	Programmes should incorporate learning of practical skills such as reading nutrition labels	Include content on healthy portion sizes healthier ways to prepare traditional foods, alongside Western foods. Hands on healthy food preparation and tasting session
Address excessive consumption of ‘junk food’ and takeaways	Psychological capability Physical opportunity	Training Enablement	19. Material/guidance based on preferences of target population 26. Intervention addresses health behaviour patterns found in target populations	Conception/ planning Implementation Outcome	Programmes should include behaviour change techniques to increase confidence and motivation in ability to make changes and also include parent skills training	Incorporate training on parenting skills, cut down on undesirable behaviours and change food availability in the home Set achievable targets and rewards
Difficulty understanding food labelling and purchasing healthy foods	Physical capability	Training	19. Material/guidance based on preferences of target population 36. Provide ethnically/culturally appropriate food/activities	Conception/ planning Implementation Outcome	Programmes should incorporate learning of practical skills such as reading nutrition labels	Educational interactive activities on food labelling Hands on healthy food preparation and tasting session
Parents’ perceived ability to influence their child’s eating behaviours	Psychological capability	Enablement Training	23. Intervention goals and outcomes are culturally appropriate 26. Intervention addresses health behaviour patterns found in target populations 38. Address emotional barriers and stressors 41. Encourage/involve social support	Conception/ planning Implementation Retention Outcome	Programmes should include behaviour change techniques to increase confidence and motivation in ability to make changes and also include parent skills training	Improved social support to encourage self-belief Incorporate parenting skills training. Set achievable healthy eating targets and rewards

^a^In Liu et al.’s Typology of cultural adaptation, each adaptation type is numbered and it is these numbers that are used in this column

^b^NICE guideline PH47:Weight management: lifestyle services for overweight or obese children and young people

### Findings from the existing first steps programme observation and consultation with the managing staff

A researcher (TG) observed two programmes, delivered by different facilitators (all sessions of one programme and two sessions of the other programme). Observations broadly concurred with the qualitative data. Particularly evident were: the lack of interactive activities for participants; the large volume of written information handed out; and the heavy focus on nutritional knowledge, with less emphasis on skills around food preparation and feeding practices, and little physical activity content. Goal setting was incorporated in the programme sessions but was not always well implemented. The programme managers also identified that the didactic delivery and volume of written information were problematic.

### Application of the behaviour change wheel and cultural adaptation theory

Through the mapping of the COM-B elements to the qualitative data, the intervention functions of enablement and education were identified as appropriate to address all target behaviours. Environmental restructuring, persuasion and incentivisation were identified as functions to address programme attendance, and training was identified as a function to address physical activity and healthy eating. Modelling was also identified as a way to address the physical activity behavioural target.

From the parallel process of mapping the 46-item cultural adaptation typology [15] to the qualitative themes, several types of cultural adaptation and the stages at which they could be applied in the programme cycle were identified. This process ensured that there was explicit consideration of how adaptations to the programme were culturally appropriate to the target population. The qualitative themes, mapped COM-B components, intervention functions, cultural adaptations and programme cycle stage, and corresponding NICE guideline recommendations are presented in Table 4.

### Detailed planning of the culturally adapted programme

Following application of the two guiding frameworks to the qualitative data, specific adaptations were planned by two members of the research team (TG and MP). This planning was also informed by the research evidence and local programme information. Further consultation with the programme managers took place at this point so that they could comment on the feasibility of delivery of the planned programme. The specific adaptations are outlined in the right-hand column of Table 4. To further illustrate how the adaptation process was undertaken, an example of the process is given in Additional file 1. When the adaptation process was completed, the planned intervention programme was presented to the Parent Advisory Panel for feedback.

### Final intervention design

A summary of modifications made as a result of the adaptation process is provided below. The adapted intervention programme is reported in more detail using the Template for Intervention Description and Replication (TIDieR) checklist [37] (see Additional file 2).

#### Programme promotion and recruitment

The initial written and verbal contact with families who were referred to the service was modified so that non-English speaking parents were contacted by telephone in their preferred language.

#### Key changes to programme structure and delivery

Session length was increased from 60 to 90 min, and provision of weekend programmes was increased. Children were included in all programme sessions. Flexibility was built into all programme sessions to enable a degree of tailoring to the individual families attending. This was achieved through the development of interactive activities that helped families identify their specific challenges and have the opportunity to discuss these with the facilitator.

#### Session content

The emphasis of the programme was changed so that there was more focus on changing eating and physical activity behaviours to improve health, and less focus on weight. Sessions were adapted to include much more interactivity, and physically active elements were introduced into every session. Content was also designed to encourage interaction and peer support between the families. Behaviour change techniques were incorporated across the programme, and a specific parenting session was developed to help parents think about how they can best support their child to change their behaviours.

#### Developed resources

Colourful visual display boards and resources for the interactive activities were developed for use within the sessions, as this was recognised as an important factor in engaging children and families. All materials were designed to have pictorial representations and minimal written information. To further encourage interactivity, a website was developed as a supporting resource for families (both parents and children). This was mainly in English, but Urdu and Bengali translations were available for the front page introduction and the Frequently Asked Questions section. A facilitator guide and two facilitator training sessions were developed.

## Discussion

The aim of this study was to adapt a selected child weight management programme to make it more relevant and acceptable to Pakistani and Bangladeshi families so that once they commenced the programme, they would be more likely to complete it. The intervention adaptation process was multistage and iterative, and was informed by the experiences and views of programme participants and providers, as well as incorporating the available research evidence relating to children’s weight management intervention.

The BCW [33] and Typology of cultural adaptation [15] frameworks enabled us to use the qualitative data to develop a theoretical understanding of the behaviour of families and how adaptations to the programme could support behaviour change whilst also being acceptable to all families. This resulted in explicit articulation of how the different elements of the programme were designed to positively influence the identified target behaviours. The cultural adaptation typology enabled a focus on cultural needs throughout the process, but it became clear that many of the adaptations required were not specific to the cultural groups that we were focusing on, and related more to addressing the daily challenges faced by families that impaired their ability to undertake healthy behaviours. It was also clear from the qualitative study and local programme information that there is a need to deliver the programme in a flexible and responsive way so that the needs of individual families are met, as family contexts differ greatly, regardless of their ethnicity. Therefore, the adapted intervention was designed to incorporate flexibility so that facilitators delivering the programme could respond to the needs of all participants. This approach is coherent with the recognised need for a conceptual shift from a traditional focus on ‘ethnic groups’ to a greater understanding of population diversification in terms of a range of related and dynamic factors linked to migration (so-called super-diversity) [38].

The adaptation process also provided opportunity to ensure that the design of the programme was informed by the current children’s weight management research evidence. We searched for all relevant literature but there was little further information to add that was not already captured in the NICE guidelines [5], which were published in November 2013 and were underpinned by two comprehensive systematic literature reviews [30, 31]. Therefore, the adaptation process incorporated an explicit step of considering any relevant NICE recommendations.

Cultural adaptation can occur at two levels: surface and deep structure adaptations. The former are adaptations which address the visible characteristics of a minority ethnic group, for example, adaptations to address language needs or including culturally matched images and foods in materials. The latter address less visible aspects such as core values and beliefs that contribute to a person’s world view [39, 40]. The adaptations made in this study addressed both levels. The responsiveness of the programme to individual family contexts, the focus on health rather than weight loss, and the fostering of peer support are all adaptations at the deep structural level.

There is still relatively little research into the cultural adaptation of health promotion programmes. In 2012 a landmark review on health promotion programme adaptation for minority ethnic groups was published. This synthesised literature on health promotion programmes targeting diet, physical activity and smoking [15], and highlighted that most research in this field is US based and focused on African-American communities, which limits the applicability of the findings to the UK context. This is reflected in childhood obesity intervention research, where the focus has been on US minority ethnic communities. Systematic reviews of culturally targeted interventions have highlighted that adaptations are often confined to the surface level, although there are some examples of deep structural adaptations [14, 41]. A lack of reporting of the adaptation strategies used has also been highlighted [41], which limits understanding of the theory which underpins the adapted programmes. A particular strength of this study is that we have used formative research and applied theoretical frameworks in our cultural adaptation approach, which has resulted in explicit articulation of the theory underpinning the adaptations made to the programme.

The study had some limitations. Recruitment to the qualitative study was challenging, with limited success in recruiting participants in the completing group to the FGs, despite efforts to make them as convenient and accessible as possible. There may be cultural reasons contributing to this non-attendance, which we have yet to identify. These may in part also contribute to high attrition from weight management programmes seen in families from these communities. The low number of participants in the FGs potentially limited the richness of the data, as group sizes of 6–8 are needed to maximise group interaction and discussion [28]. However, even with the limitations of the FG data, we were still able to identify differences between the completing and non-completing/non-attending families (e.g. perceptions of their child’s weight as a problem, expectations around gaining new knowledge from programme attendance etc.). Another potential limitation is that despite explaining the nature of the research, and that it was being undertaken by an independent organisation, some participants still believed the research team to be part of the children’s weight management service, which may have influenced the data obtained in the study. For example, they may have been less willing to be critical of the programme. Even taking into account these limitations, we were able to collect rich data that yielded valuable information that fed into the adaptation process.

It is possible that adapting the programme to suit the specific needs of Pakistani and Bangladeshi families could be discordant with families from other cultural communities. However, many of the issues raised by parents within this study are coherent with the wider literature on barriers and facilitators to families attending weight management programmes [42]. In addition, flexibility to respond to different family contexts was incorporated into the adapted intervention, which enables a degree of tailoring to all families. The subsequent feasibility trial of this culturally adapted intervention that we have undertaken and reported in a separate paper [20] gives further information on the acceptability of the programme to Pakistani and Bangladeshi families, and families who are not from these communities.

## Conclusions

In this paper we have presented a process of cultural adaptation of a children’s weight management programme, which has resulted in a programme that is culturally adapted at both the surface and deep structural levels. The process undertaken has enabled us to explicitly articulate the theory which underpins the adaptations that have been made. The theoretical approach that we used could potentially be replicated by others who are planning to culturally adapt health promotion programmes.

## Additional files

Additional file 1: CHANGE study: an example of the intervention adaptation process. (DOCX 14 kb)

Additional file 2: The CHANGE study adapted children’s weight management intervention: Template for Intervention Description and Replication (TIDieR) checklist. (DOCX 18 kb)

## Data Availability

All data are available on request from the corresponding author.

## Abbreviations

BCW: Behaviour Change Wheel
BMI: Body Mass Index
CHANGE: Child weigHt mANaGement for Ethnically diverse communities
COM-B: Capability, Opportunity, Motivation and Behaviour
FG: Focus Group
MRC: Medical Research Council
NHS: National Health Service
NICE: National Institute for Health and Care Excellence
RCT: Randomised Controlled Trial
TIDieR: Template for Intervention Description and Replication
UK: United Kingdom
USA: United States of America
