# Supplementary material for: Cultural adaptation of a children’s weight management programme: Child weigHt mANaGement for Ethnically diverse communities (CHANGE) study
Source: BMC Public Health. 2019 Jun 28;19:848. doi: 10.1186/s12889-019-7159-5 (PMC6599293; doi:10.1186/s12889-019-7159-5)
Supplement: Supplementary file 1 — CHANGE study: an example of the intervention adaptation process. (DOCX 14 kb) [file 12889_2019_7159_MOESM1_ESM.docx]

**CHANGE study: an example of the intervention adaptation process**

***Theme arising from qualitative data:*** parents perceived that they lacked the ability to positively influence their children to undertake physical activity.

***Application of the Behaviour Change Wheel (BCW):*** we identified through the COM-B model that parents' psychological capability needed to be addressed, and that this could best be achieved through the intervention functions of enablement and training.

***Application of the Typology of cultural adaptation and programme theory relating to health promotion programmes:*** we identified types of adaptations that may address this lack of psychological capability. These were: making intervention goals culturally appropriate; addressing health behaviour patterns found in target populations; addressing emotional barriers and stressors; and encouraging social support. We also identified that adaptations should be considered at various stages of the programme cycle (planning, implementation, retention and outcome stages).

***Reference to NICE guideline:*** we identified a relevant NICE guideline recommendation: 'Programmes should include behaviour change techniques to increase confidence and motivation in ability to make changes and also include parent skills training', and incorporated it into our adaptation planning.

***Development of specific adaptations:*** following the above processes and taking into account information from our direct observation and consultation with the weight management providers, we planned specific adaptations to the programme, incorporating evidence based behaviour change techniques if appropriate. The specific adaptations to address parents’ perceptions of their lack of ability to positively influence their children in undertaking physical activity were: increasing social support to encourage self-belief (enablement); encouraging parental physical activity (enablement); incorporating parenting skills training (training); and setting and reviewing achievable and culturally appropriate targets relating to their child’s physical activity (enablement).
