# Supplementary material for: Cultural adaptation of a children’s weight management programme: Child weigHt mANaGement for Ethnically diverse communities (CHANGE) study
Source: BMC Public Health. 2019 Jun 28;19:848. doi: 10.1186/s12889-019-7159-5 (PMC6599293; doi:10.1186/s12889-019-7159-5)
Supplement: Supplementary file 2 — The CHANGE study adapted children’s weight management intervention: Template for Intervention Description and Replication (TIDieR) checklist. (DOCX 18 kb) [file 12889_2019_7159_MOESM2_ESM.docx]

**The CHANGE study adapted children’s weight management intervention: Template for Intervention Description and Replication (TIDieR) checklist**

| **1: Name** | ***First Steps***. The name of the current child weight management programme in Birmingham was retained, ensuring that in the feasibility study the comparator and intervention programmes had identical titles so that participants would be unaware of whether they were attending a standard or adapted programme. |
| --- | --- |
| **2: Why** | The primary aim of adapting the intervention programme was to increase acceptability and value of the programme to families from Pakistani and Bangladeshi communities in order to increase their likelihood of completing the programme, whilst maintaining acceptability of the programme to families of other ethnicities. In addition, the adaptation process was an opportunity to ensure the programme was consistent with current evidence.  The adaptation process was informed by data from Pakistani and Bangladeshi families of overweight children, current literature and local programme provider experience and was guided by two theoretical frameworks (BCW and Typology of cultural adaptations). The frameworks ensured that factors influencing behaviour that required change were identified and targeted, and the adaptations made were culturally appropriate to the Pakistani and Bangladeshi communities. Adaptations were made to the structure, delivery and content of the programme. |
| **3 & 4: Materials and Procedures** | Families referred to the children's weight management service are sent a letter inviting them to attend their nearest children's weight management programme. This initial invitation letter was redesigned to be more engaging and emphasise the aims and benefits of the programme. All families receiving an initial invitation letter will also have a follow up phone call and this should be in their preferred language.  The intervention programme consists of six 90 minute sessions, delivered over six weeks. Both children and their parents/carers attend all sessions. Programmes run on weekdays (50% of programmes) or on Saturdays (50% of programmes). A step by step programme delivery guide was developed for the programme facilitators, with a training programme alongside, comprising a 2.5 hour session and a follow up 1.5 hour session.  Attractive programme materials were developed for use within the programme, including visual display boards, and materials for the interactive components. At the first session, participants receive a First Steps branded ring binder in which to keep their goal sheets and activity sheets that are given each week. Non-English speaking participants are accompanied by an interpreter. Attendance at each programme session is rewarded by a gold star on the participant's attendance sheet. Progress towards behavioural goals are also rewarded with gold stars. A website was developed for use by participants, which provides a variety of resources, including recipe and physical activity ideas, frequently asked questions, worksheets and games, and links to other relevant resources.  Aims and outlines of the six programme sessions are as follows:  *Week 1: Welcome*  Aims   - Provide a welcoming, friendly supportive environment - Improve knowledge on why a healthy lifestyle is important - Collect baseline height and weight data   Outline   1. Welcome activity: fruit and vegetable jigsaw 2. Display board 1: welcome to First Steps, hand out folders, explain attendance rewards 3. Activity: icebreaker (chosen by facilitator) 4. Display board 2: What to expect 5. Display board 3: First Steps goals 6. Display board 4 and Activity: participants working together to identify goals 7. Activity: parents and children record their personal goals for the programme 8. Height and weight measurements taken during last activity 9. Physical activity: get people moving and socialising 10. Introduce website   *Week 2: Healthy eating*  Aims   - Provide basic information on healthy eating - Improve knowledge on why a good diet is important for health - Learn about making simple changes   Outline   1. Physical activity: active game to get people moving 2. Display board 1: healthy eating-striking a balance 3. Activity: healthy and unhealthy eating habits cards 4. Display board 2: what should we be eating? 5. Display board 3: sugar fat and salt 6. Display board 4: snacking 7. Activity: parents and children identify healthy/unhealthy snacks in their own diets 8. Display board 5: portion sizes 9. Display board 6 and activity: setting healthy eating goals   *Week 3: Making changes*  Aims   - Review healthy eating goals - Create a supportive and encouraging environment for families to discuss aspects of behaviour change they have struggled with - Provide parents with ideas and suggestions of how to encourage behavioural changes within their family - Enable children to undertake fun physical activities   Outline   1. Review of progress towards healthy eating goals and rewards 2. Children leave to attend a separate one-hour fun physical activity session 3. Display board 1: changing family eating and activity habits 4. Display board 2: tools to support changing behaviours of children 5. Display board 3: coping with children's resistance to change 6. Display board 4 and activity: case discussions on approaches to changing unhealthy behaviours 7. Activity: parents identify strategies to cope with their child's unhealthy behaviours   *Week 4: Physical activity*  Aims   - Review parents' progress towards behaviour goals - Provide basic information on physical activity - Improve knowledge on why physical activity is important - Provide ideas and strategies for getting families active   Outline   1. Review with parents the goals set related to their child's behaviour 2. Physical activity: active game to get people moving 3. Display board 1: why is physical activity important? 4. Display board 2: how to get active 5. Display board 3: time to stop making excuses 6. Activity: completing a physical activity timeline 7. Display board 4 and activity: setting physical activity goals   *Week 5: Give it a go*  Aims   1. Review physical activity goals 2. Learn about food labelling 3. Provide families an opportunity to work together making healthy snacks   Outline   1. Review progress towards physical activity goals and reward 2. Rotation around the following activities:  - Food labelling and food shopping card - Milk labelling - Drinks Labelling - Health food preparation station   *Week 6: Review and celebrate*  Aims   - Celebrate the end of the course and the achievements made by the group - Review programme goals set in week 1 - Encourage participants to continue with and sustain lifestyle changes - Collect end of programme height and weight data   Outline   1. Review family progress towards programme goals and reward 2. Activity: healthy lifestyle quiz 3. Activity: setting sustainable family targets 4. Height and weight measurements taken during activities 5. Evaluation forms completed 6. Award certificates of completion 7. Physical activity: active game to get people moving |
| **5: Who provided** | Two employees in the service provider organisation were trained to deliver the adapted programme. They both had a minimum of 12 months experience of delivering a children's weight management programme. A third employee was trained to assist in delivery, in particular, supporting the interactive activities, running the children's physical activity session in week 3 and collecting height and weight data. The facilitators were provided with a detailed delivery guide and two training sessions, which were run by the CHANGE study research team. |
| **6: How** | The programme is designed to be delivered to a group of families (optimum group size is 10 families). |
| **7: Where** | The programme is designed to be delivered in local community venues (e.g. community centres or primary schools). |
| **8: When and how much** | The programme is delivered through six 1.5 hour sessions over six weeks. A higher proportion of programmes were planned to be delivered on Saturdays than the standard programme. |
| **9: Tailoring.** | Throughout the programme, there is provision for tailoring to individual family needs. This is built into the planned interactive components and the facilitator delivery guide |
